# A Data-Driven Expectation Prediction Framework Based on Social Exchange Theory

**DOI:** 10.3389/fpsyg.2021.783116

**Published:** 2022-01-10

**Authors:** Enguo Cao, Jinzhi Jiang, Yanjun Duan, Hui Peng

**Affiliations:** ^1^Intelligent Interaction Design Laboratory, School of Design, Jiangnan University, Wuxi, China; ^2^School of Art and Design, Zhengzhou University of Light Industry, Zhengzhou, China

**Keywords:** social exchange theory, expectation prediction framework, user comment, user requirement analysis, data-driven design

## Abstract

Along with the rapid application of new information technologies, the data-driven era is coming, and online consumption platforms are booming. However, massive user data have not been fully developed for design value, and the application of data-driven methods of requirement engineering needs to be further expanded. This study proposes a data-driven expectation prediction framework based on social exchange theory, which analyzes user expectations in the consumption process, and predicts improvement plans to assist designers make better design improvement. According to the classification and concept definition of social exchange resources, consumption exchange elements were divided into seven categories: money, commodity, services, information, value, emotion, and status, and based on these categories, two data-driven methods, namely, word frequency statistics and scale surveys, were combined to analyze user-generated data. Then, a mathematical expectation formula was used to expand user expectation prediction. Moreover, by calculating mathematical expectation, explicit and implicit expectations are distinguished to derive a reliable design improvement plan. To validate its feasibility and advantages, an illustrative example of CoCo Fresh Tea & Juice service system improvement design is further adopted. As an exploratory study, it is hoped that this study provides useful insights into the data mining process of consumption comment.

## Introduction

The continuous digitization of the consumption field requires the transition of the retail business framework from physical sales to a new service system for online and offline sales (Brenner et al., 2014). Meanwhile, the changes in consumer behavior influenced by the Internet and social media technologies (Williams et al., 2008), purchase intention of customers, and demand experience have become the focus of consumer-centered design research (Dash et al., 2021). New technologies such as data mining (Chiu and Lin, 2018) and digital twinning (Tao et al., 2018) greatly assist the value co-creation between enterprises and users (Peltier et al., 2020).

Conventional demand analysis techniques, such as user interviews, fieldwork, or focus group interviews, are time consuming and labor-intensive with limited research scope and are no longer suitable for user demand mining in the field of interconnected product service systems (Song, 2017). Consumers with different backgrounds, consumption habits, and preferences are spread all over the world (Koitz and Glinz, 2018), which makes it more difficult to quickly identify stakeholders and their needs. Confronted with this problem, the user comment data collected from online network platforms have contributed greatly to the realization of more efficient and timely user demand mining, in the meanwhile, the success of the product-service system in the digital service economic background relies much on the quality of user-generated data analysis (Zheng et al., 2020). In consequence, there is a need to develop a systematic framework for user requirement analysis and expectation prediction to better guide comment data analysis and demand mining.

Based on the social exchange theory, this study establishes a framework to guide the expectation prediction process by using massive user comment data to analyze the exchange demands of consumers. Specifically, we discussed the concept and definition of the product, service, information, and other exchange resources involved in the consumption exchange process and argued that it is suitable for guiding user comment data classification in the consumption field. Then, we proposed a specific data analysis process to mine and predict the consumption expectations of users on various exchange resources. In summary, based on the social exchange theory, two key challenges for constructing a data-driven expectation prediction framework are to (1) obtain massive amounts of available data from online platforms and mine effective information and (2) define the consumption exchange elements and expand expectation prediction to the consumption and use stage.

The rest of this study is organized as follows. A holistic review of related works is given in the “Literature review” section. The development process of the expectation prediction framework is represented in “The proposed expectation prediction framework” section. An illustrative example of CoCo Fresh Tea & Juice service system design improvement is given to validate the feasibility of the proposed framework in “An illustrative example” section. The advantages and limitations of the framework and example are further discussed in the “Discussion” section. In the “Conclusion” section, contributions and future work are summarized.

## Literature Review

This section summarizes the basic notions of the social exchange theory and gives a comprehensive review of the recent research of data-driven design, as well as its tools/methods to enable such expectation prediction framework development.

### Basic Notions of Social Exchange Theory

Social exchange theory focuses on the exchange behavior between people (Zolle and Muldoon, 2019), which has been supplemented and expanded by many scholars in management, sociology, social psychology, and other fields (Boateng et al., 2019). After combining the relevant theories of social exchange, Roloff (1981) pointed out that social exchange includes three viewpoints, social exchange theory suggested by Homans (1950), resource exchange theory suggested by Foa and Foa (1974), and equity theory suggested by Walster et al. (1976). Among them, the social exchange theory of Homans and the resource exchange theory of Foa are the main theoretical reference bases of this study.

Homans focused on personal interests (Muldoon et al., 2018) and believed that individuals will rationally weigh the benefits and the cost of their exchange behaviors (Boateng et al., 2019). In other words, the expectation of individuals of behavior reward can determine the likelihood of behavior. As a result, the attitudes or behavioral intentions of users are predictable (Hamari et al., 2016). Social exchange theory has been used to explain social media platform interaction (Surma, 2016), sharing motivation of consumers (Ham et al., 2019; Wang et al., 2019), and reciprocal behavior (Tsai and Kang, 2019). It has also been used to explore trust (Colquitt et al., 2014) and data privacy (Li et al., 2016). For example, the perceived value of users is conceptualized as a multidimensional construct with cognitive and affective aspects (Dewi et al., 2020). Based on the principle of reciprocity to explore the impact of social influence on sustainable consumption behavior of customers (Wang et al., 2019).

While Homans regarded social exchange behavior as the exchange of material and non-material commodities (Treviño, 2009), Foa divided social exchange resources into six categories: love, status, service, information, commodity, and money (Cooper-Thomas et al., 2018; James et al., 2021). They refined the concept and classification of exchange resources and pointed out that individuals tend to exchange similar resources (Roloff, 1981), for example, it is easier to exchange money for products than to exchange love for money (James et al., 2021). In the context of the digital service economy, the interactive forms and exchange contents of the participation of users in consumption exchange have become richer. Many scholars pointed out that the factors affecting the purchase experience of consumers generally include economic benefits, service quality, information transmission, social emotions, product functions, and perceived value (Muthusamy and White, 2005; Mohlmann, 2015; Zhu et al., 2017). Pallant et al. (2022) pointed out that social exchange theory is a useful framework to understand user-generated data toward consumer-retailer exchange. By adapting the research of Yang et al. (2015) obtained six attributes, namely, visual beauty, navigation, entertainment, community-driven, privacy and security, and user-friendliness, and regarded them as the driving factors for users to participate in social shopping websites.

**FIGURE 1:**
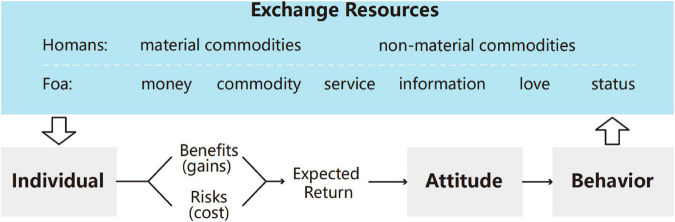
Social exchange behavior and resource classification.

Based on the above theoretical research, the social exchange behavior and resource classification are summarized, as shown in Figure 1. Individuals measure the benefits (gains) and risks (costs) (Heo and Chang, 2018) of exchange resources, estimate behavioral rewards, and then generate exchange willingness and exchange behavior. The level of expected return will directly determine the strength of the willingness of an individual to exchange, which, in turn, affects the possibility and sustainability of the exchange behavior (Chou and Hsu, 2016). Zhou and Jin (2009) summarized “success,” “stimulation,” and “value” propositions of Homans and concluded that the possibility of behavior is equal to the value multiplied by the probability.

### Related Works and Methods in Expectation Prediction

**FIGURE 2:**
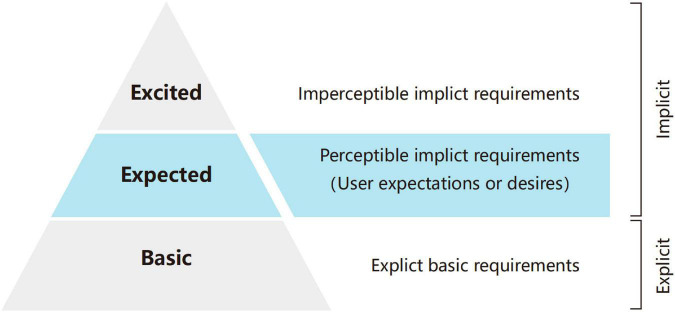
Three layers of user requirements.

User expectation prediction can be regarded as a process of capturing the expectation of stakeholders based on related resources (Li R. et al., 2020), mining implicit requirements, and predicting the trend of demand change (Song, 2017). User expectations are implicit and non-verbalized requirements (Soren and Otto, 2001). Luo and Lu (2006) divided user requirements into the basic layer, expected layer, and excited layer (shown in Figure 2) according to the Kano model (Kano, 1984; Luo and Lu, 2006). The basic layer represents explicit basic requirements and requests the basic functions must be satisfied. The requirements in the expected layer and excited layer are implicit requirements, which stem from the inability of existing functions (Luo and Lu, 2008) to achieve higher satisfaction of users. The requirements in the excited layer are hidden in the subconscious of users, while in the expected layer, users understand what their unsatisfied requirements are and then generate personal expectations or desires. Scholars usually identify the explicit and implicit needs of users through the original data from users (Lee et al., 2019) and emphasize that user satisfaction can be greatly improved when implicit demand is satisfied (Yongtai, 2007).

As mentioned earlier, the expectations of users can be predicted through user evaluation of exchange resources, while a large number of online comment data (Jin et al., 2016) can effectively reflect the satisfaction evaluation of users on the consumption resources. As for the requirement analysis methods, two types of methods are mainly applied, including traditional research methods, such as the KJ method, user experience journey map, empathy map, and data analysis methods, such as machine learning algorithm and cluster analysis (Kim and Jun, 2015; Yu et al., 2020). For example, clustering algorithms are used to analyze web log data (Tang et al., 2020; Wen et al., 2020) (such as purchase records and online behaviors) and word frequency statistics are used to extract keywords in user comment texts (Zhao et al., 2019; Zhu et al., 2020). Among them, Li N. L. et al. (2020) proposed a model to identify key demands and used text mining and clustering algorithm to analyze online user comments. Chiu and Lin (2018) used text mining and Kansei engineering to extract customer preferences to predict the future trend of target consumer objects. Jiang et al. (2019) extracted time-series data from online comments for customer dynamic preference analysis. Liang et al. (2016) applied the law of demand evolution to predict the functional needs of users for the target products. Lai et al. (2019) mined implicit requirements of users from dynamic Internet data. Lee et al. (2021) used the semantic lexicon to analyze the emotions of users about products and services in online comments of consumers.

On this basis, expectation prediction is more inclined to make use of the predictive characteristics of data analysis, based on a large amount of data to dig hidden demands of users and make relevant predictions. This research focuses on the analysis of user requirements in the expected layer and predicts user expectations through big data analysis methods.

## The Proposed Expectation Prediction Framework

**FIGURE 3:**
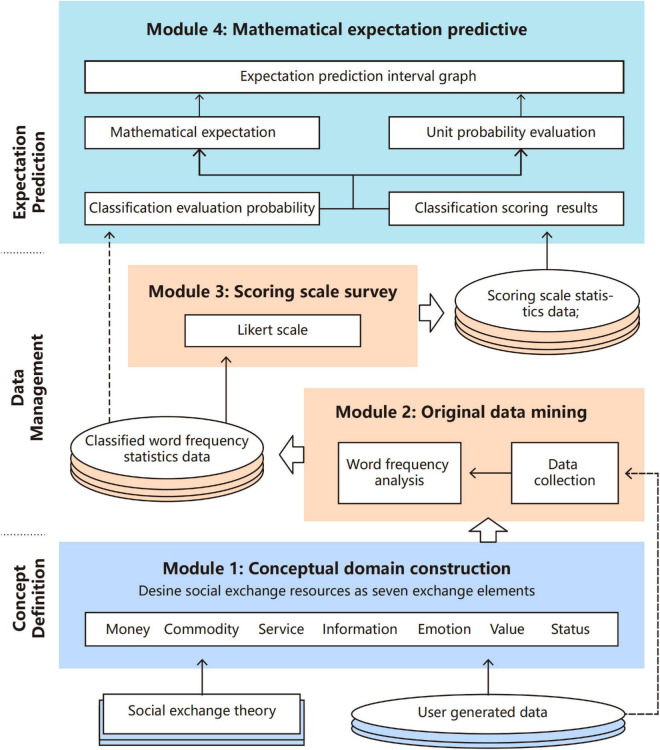
Data-driven expectation prediction framework.

This section proposes a data-driven expectation prediction framework based on the social exchange theory, as shown in Figure 3. From the point of view of system architecture, the framework mainly includes three layers, namely, the concept definition layer, data management layer, and expectation prediction layer. (1) Concept definition layer aims to define the classification standards of the exchange elements in the framework. With reference to user-generated data of target objects, the consumption exchange elements are organized and classified in a clear concept based on social exchange theory. (2) In the data management layer, data such as web service logs and user comment text constitute the original data resources. For the proposed expectation prediction framework, different user-generated data can be collected according to the research object. The data management process is as follows: collect data, use word frequency statistics, use Likert scale survey, and obtain target dataset. (3) Data management layer provides data support for predictive analysis to the expectation prediction layer. The expectation prediction layer describes the process of applying mathematical expectation algorithm and statistical analysis methods to construct an expectation prediction interval graph and finally predict the expectation trend of users.

In order to construct the framework, the following four core modules including conceptual domain building module, original data mining module, scoring scale survey module, and mathematical expectation prediction module are further elaborated below.

### Module 1: Conceptual Domain Construction

**FIGURE 4:**
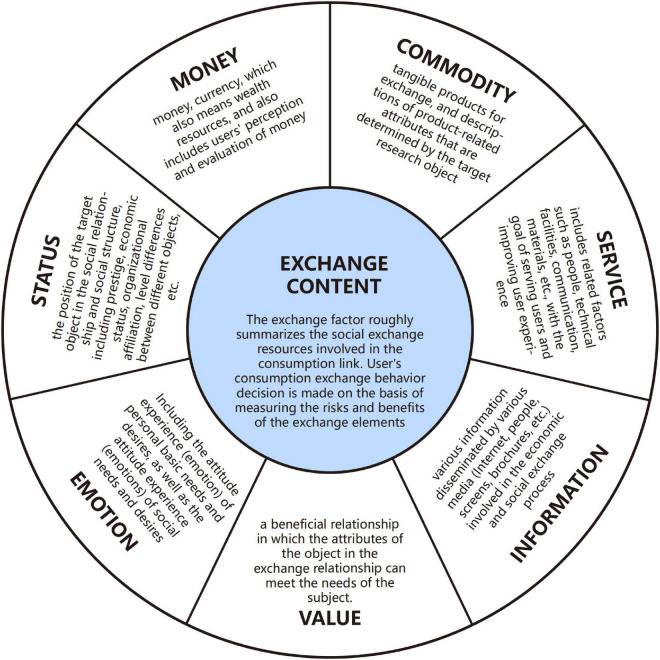
Social exchange content block diagram.

The main purpose of Module 1 is to clarify the specific content of social exchange resources in the consumption process and define the concept of the exchange elements domain. Consumption behaviors of users are not only affected by economic benefits at the material transaction level but also affected by personal perception preferences. As mentioned earlier, exchange resources have been classified into six categories: money, commodity, service, information, love, and status. “Money” and “commodity” are generally recognized as material exchange resources (Mohlmann, 2015). “Service” includes service personnel, service facilities, and intangible service system, so it is both material and non-material resources. Non-material resources can be mainly summarized as “emotion,” “information,” “value,” and “status” (G. Zhu et al., 2017). Compared with the “love” proposed by Foa, “emotion” is more suitable to describe that the emotions and feelings exchanged in the consumption process (Lawler, 2001). “Information” also runs through the entire exchange process. Meanwhile, individuals will produce additional value perception in social exchange, such as cultural value, situational value, and social value (Olsen, 2015). Therefore, “value” is also a part of the exchange elements. Finally, the concepts of social approval, authority (Liao, 2008), and hierarchy in social exchange rewards can all be summarized as “status.” Based on this, this study summarizes exchange elements into seven domains: money, commodity, service, information, value, emotion, and status, as shown in Figure 4.

The division and definition of these seven concepts are based on the analysis of rigorous user-generated data and other relevant studies, have a sufficient theoretical basis, and fit the reality truly and effectively. Therefore, it has a certain basis and rationality for division. In the meantime, in the expectation prediction framework, the statistical results of tag word classification and the survey results of the scoring scale both can verify the feasibility of the concept definition.

### Module 2: Original Data Mining

#### Data Collection

The original data resources come from user-generated data in online social network services, including service log data and user comment data (Olmedilla et al., 2016). Massive user-generated content has extremely high mining and utilization value. Compared with service log data, user comment data are easier to obtain on web pages where the data are disclosed (Khan et al., 2020). User comment data usually include numbers, review text, and images (Hossin et al., 2019). This study focuses on digging out semantically meaningful concepts in user comment texts, making them a powerful data resource for predicting user expectations. This study mainly uses web mining techniques (Lee and Shiu, 2004) to obtain comment data in the target web page and preprocess the data to form a user comment database.

#### Word Frequency Analysis

As an important means of text mining, word frequency analysis (Kim and Jun, 2015) can count the occurrence times of important words in the text and determine the hot spots and trends of changes (Dicle and Dicle, 2018). With the popularization of data mining methods, many online word frequency analysis websites and word frequency analysis software have appeared. This research imports the collected user comment database into word segmentation software and makes preliminary sifting according to the part of speech and word frequency, such as removing keywords with a word frequency of <3; removing duplicate words and merging synonyms; and extracting nouns, adjectives, verbs, adverbs, and pronouns to form a candidate collection.

Keywords were filtered manually and classified into each element category according to the conceptual domain constructed in Module 1. Then, the classified word frequency statistics data are obtained, and classified evaluation probability is equal to the classified word frequency divided by the sum of total word frequency, which is one of the key datasets for mathematical expectation calculation in Module 4. In addition, the conceptual domain construction in Module 1 can also be adjusted and optimized according to the feedback from the keyword classification result.

### Module 3: Scoring Scales Survey

During the second survey, a method based on the Likert scale was proposed. The scale question bank was divided into seven dimensions according to the classification of exchange elements. Referring to the statistical data of classified word frequency, some high-frequency keywords were integrated into each exchange element category to get the scale sentences. The Likert scale is a common scale tool in the field of social sciences (Matas, 2018), which is often used to measure opinions or satisfaction. To get the evaluation attitudes of users to exchange elements, this study uses a scoring scale to collect scoring scale statistics data and calculates classification scoring results of users. The scoring scale requires subjects to express their personal views on a series of scale sentences. According to the Likert scale template, each statement generally has five levels of answer options (Nam, 2019), such as “very satisfied (5),” “satisfied (4),” “not necessarily (3),” “unsatisfied (2),” and “very dissatisfied (1).” The level of the scale can also be determined according to the specific situation. We used a 6-level scale to avoid the tendency of subjects to choose the intermediate option of “not necessarily.” The specific design process of the scale includes question bank compilation, expert evaluation, question design, data collection, and analysis and testing. Finally, the data collected from the scale are integrated into scoring scale statistics tablet as the variable of mathematical expectation calculation in Module 4.

### Module 4: Mathematical Expectation Prediction

**FIGURE 5:**
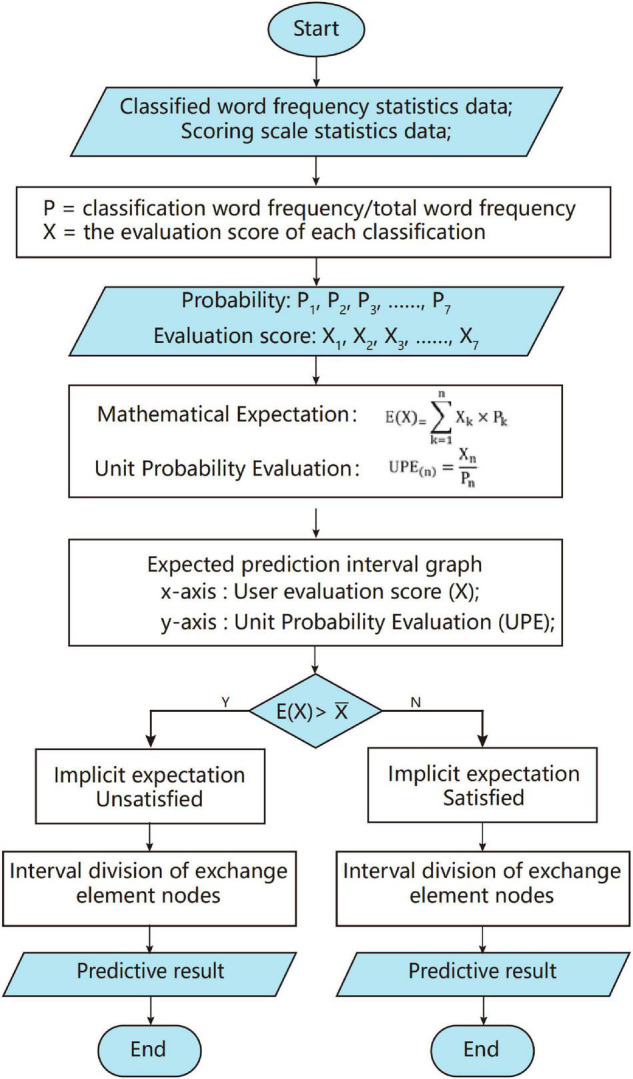
Expectation predictive process.

Social exchange theory defines the product of reward value and its acquisition probability as the possibility of user exchange behavior, which corresponds to the variables and probability of the mathematical expectation formula. Therefore, this study uses mathematical expectations to calculate the consumption expectation of users. The essence of expectation prediction is to explore the expectations of users for each exchange element based on the user evaluation value and appearance probability of each field. The expectation prediction process is shown in Figure 5.

#### Expectation Prediction Calculation

The classified word frequency statistics data output by Module 2 and the scale score data output by Module 3 were extracted, then the classification evaluation probability and classification scoring results were calculated, respectively. The probability (*P*) of each exchange element is equal to the sum of classified word frequency divided by the total word frequency. The evaluation score (*X*) is equal to the average score of classified scale items. After acquiring the probabilities of seven elements and the corresponding user evaluation scores, the mathematical expectation and the unit probability evaluation (*UPE*) can be calculated by substituting formulas.

The mathematical expectation is the sum of the product of each variable value in the experiment and the occurrence probability of that value. It can predict the objective expected value of variables, for example, statistics of different income results of a certain event (purchasing a lottery ticket). Comparing the initial value and the expected value can help to recognize whether the event is “worth doing.” *UPE* is a newly defined quantitative indicator in this study, which represents the ratio of the evaluation score to the occurrence probability of the score, that is, the evaluation score in the unit probability. It indicates the improvement necessity of each exchange element: the higher the evaluation score, the higher the user satisfaction, the smaller the room for improvement; the lower the probability, the lower the concern of users. Therefore, the higher the *UPE*, that is, the improvement necessity is lower. The discreteE(X) function and *UPE* formula are as follows:

$$E\,\left(X\right)=X_{1}\times P_{1}+X_{2}\times P_{2}\,\cdots \,X_{n}\times P_{n}=\sum_{k=1}^{n}X_{k}\times P_{k}$$

$$U\,P\,E_{\left(n\right)}=\frac{X_{n}}{P_{n}}$$

#### Expectation Prediction Interval Graph

**FIGURE 6:**
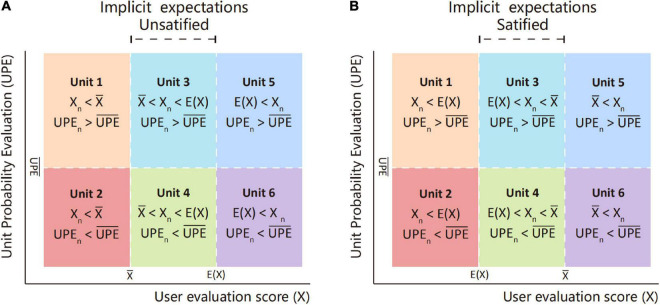
Expectation prediction interval graph of implicit expectations unsatisfied or satisfied.

As shown in Figure 6, the expected prediction interval graph is established, in which the *x*-axis represents the evaluation score (*X*) and the *y*-axis represents *UPE*. It is divided into six areas by the average evaluation score $\bar{X}$the *E*(*X*), and the average of *UPE* ($\bar{U\,P\,E}$) The arithmetic mean represents the average level of the current state, while the expectation value represents the average expected level that can be achieved theoretically. Although the average evaluation value will be infinitely close to the expectation value under the calculation of a large amount of sample data, in general, the average evaluation value will always be less than or greater than the expectation value.

On the one hand, the lower the user evaluation score (*X*) of an element, the more room for improvement. (1) When *X*_*n*_ $<min(\bar{X}$,*E(X)*), the actual evaluation of users is lower than the average and expected values, indicating that the user satisfaction with this element is far below the average or expected level, so the exchange element has a large improvement room. (2) When $X_{n}>\max\left(\bar{X},E\,\left(X\right)\right)$, the element has exceeded the average expectation level of users, and the design maturity of related content is relatively high, so there is less room for improvement. (3) There is also a situation when the value of *X* is between $\bar{X}$ and *E(X)*, that is, *X* is in the implicit expectation interval. According to the conceptual description in section “Literature Review,” the implicit expectation is between the expectation layer and the excitement layer and is the expectations that users are more difficult to perceive by themselves. At this point, when $E\,\left(X\right)>\bar{X}$, the average level of the current evaluation has not yet reached the expected level, the implicit expectation is unsatisfied (see in Figure 6A), and there is implicit room for improvement of user satisfaction. Otherwise, the implicit expectation is satisfied (see in Figure 6B).

On the other hand, the lower the *UPE* of the exchange element, the greater the improvement necessity. When *UPE*_*n*_ $<\bar{U\,P\,E}$, the improvement necessity of users is higher than the average level, then improving this element is more suitable for the preferences of most users, and user satisfaction can be improved in a larger range. When *UPE*_*n*_ $>\bar{U\,P\,E}$, the improvement of the elements causes a relatively small increase in user satisfaction.

It can be seen from the above that the exchange elements in Units 1 and 2 have a higher priority improvement, while the improvement in Unit 2 can improve user satisfaction better than Unit 1. The area of Unit 3 and Unit 4 is the implicit expectation interval. When the implicit expectation is unsatisfied ($E\,\left(X\right)>\bar{X}$), the exchange elements corresponding to this interval are highly hidden and have certain promotion room and the necessity for improvement. At the same time, compared with Unit 3, improvements of Unit 4 are more likely to produce unexpected optimization effects. When the implicit expectation is satisfied ($E\,\left(X\right)>\bar{X}$), Units 3 and 4 are similar to Units 5 and 6. The exchange elements have already met the basic expectations of users, and the room and necessity for further improvement are relatively low.

## An Illustrative Example

Aiming at the above framework and the expectation prediction process, an illustrative example of CoCo Fresh Tea & Juice service system improved design was discussed. Later, buying drinks is becoming an increasingly common behavior and is fully integrated into the lifestyle of an individual. Therefore, more and more people choose a more convenient and faster way to purchase beverages that suit their needs. This example was based on the open data of a beverage brand CoCo collected on a Chinese comment website. This brand has beverage sales channels in physical stores or online platforms and has a certain brand influence and sales position in China.

### Conceptual Domain Construction

**FIGURE 7:**
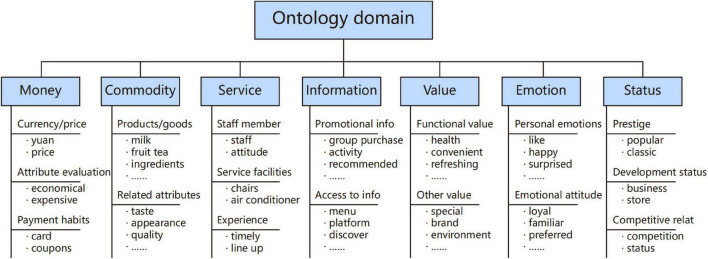
Domain ontology of beverage consumption exchange element.

According to the statement in section “The Proposed Expectation Prediction Framework,” the conceptual definitions of the seven exchange elements have been determined. Data mining and web crawler methods are utilized while extracting meaningful keyword concepts and constructing domain ontology. Since the target website contains a wealth of consumer service information, seven domain ontologies can be initially constructed based on the structure of the comment page. Meanwhile, each exchange element is further supplemented and subdivided based on the user-generated comment data and other information; in addition, the hierarchical structure of each element subcategory is discussed by service system designers. Finally, the domain ontology of the beverage consumption exchange element is established, as shown in Figure 7.

### Original Data Mining

#### Data Collection

In this example, the original data resources were mainly crawled from user comment data of the target website, including user ratings, purchase experience, and other related comments, which were represented by unstructured text and numbers. Due to the large consumer population of China and the huge beverage sales market, there are dozens of CoCo Fresh Tea & Juice stores on the target website with a large number of user reviews. Finally, six representative stores located in three types of communities (school residential area, business district, and tourist attractions) were selected. This research used a web crawler tool to assist the data collection process based on the target website. All user comments were stored in the user comment database of the CoCo in text form. A total of 2,155 user comments have been collected.

#### Word Frequency Analysis

**FIGURE 8:**
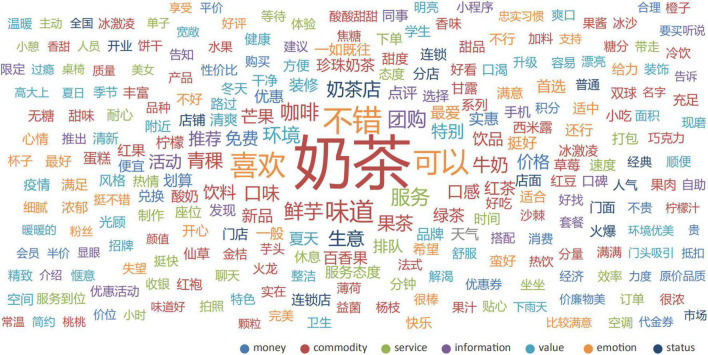
Word frequency statistics cloud map.

**FIGURE 9:**
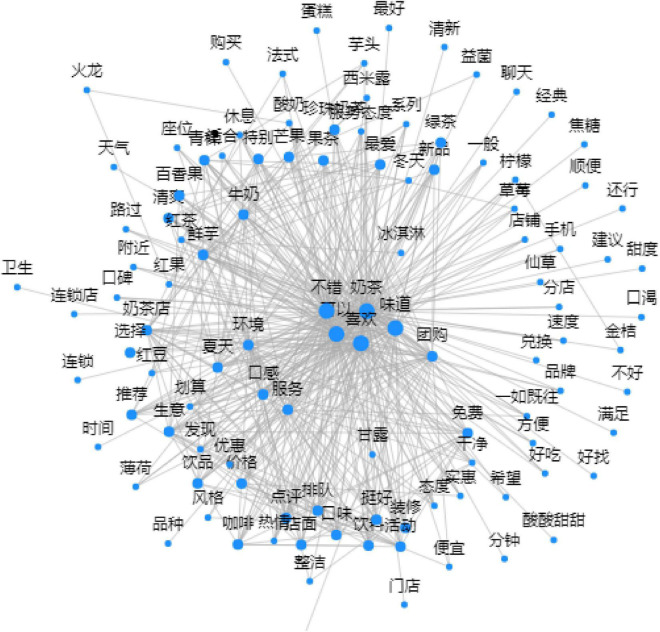
Keywords social network map.

The word frequency statistics materials were obtained from the user evaluation database of CoCo established earlier. Text data were imported into GooSeeker^1^ software for vocabulary segmentation and intelligent classification, and initially 5,555 words were obtained. Words with word frequency <3, and meaningless vocabularies such as pronouns and adverbs were eliminated, then the word frequency cloud map and social network map were derived (see Figures 8, 9). After merging synonyms or similar semantic vocabulary, 142 tags were finally created and classified according to the beverage consumption exchange element domain ontology established above. The final statistical results of the total classified word frequency and the classified evaluation probability are shown in Table 1.

**TABLE 1:** Tag words classification and statistical table.

Domain	Tag and frequency	Total	P (%)
Money	Price 138, economical 97, discounts 77, consume 39, cost performance 28 …	571	4.80
Commodity	Milk tea 1,668, ingredients 892, good taste 550, taste 439, milk 254 …	5045	39.98
Service	Service 312, line up 145, attitude 93, take-out 91, rest 82 …	1092	8.62
Information	Group purchase 241, free 201, activity 168, recommended 150, choose 102 …	1070	8.45
Value	Special 185, decorate 141, brand 87, suitable 68, refreshing 65 …	771	6.09
Emotion	Good 2,054, like 602, comment 133, favorite 132, general 90 …	3398	26.83
Status	Milk tea shop 232, business 216, store 73, classic 42, chain 42 …	752	5.94

The statistical results show that the probability of the commodity domain is the highest, the emotional domain is the second, the probability of other domains is similar, and the probability of the money domain is relatively the smallest. The social network map of keywords also shows that in the public consumer comment webpages, users are most likely to mention the products they have purchased and make corresponding evaluations. At the same time, users also tend to express personal emotional preferences directly in comments. It is worth mentioning that in the social network, many keywords co-occur with contextual elements such as seasons, weather, and neighborhood, indicating that external environmental elements will also have a certain impact on user perception during the exchange process.

### Scoring Scale Survey

The scoring scale sets seven dimensions based on seven exchange elements, and each dimension sets 3–4 evaluation items based on the tag words of the corresponding domain. A total of 100 statements were sorted out to consult the satisfaction evaluation of users on the consumption experience of CoCo in seven dimensions, such as money, commodity, and services. After expert evaluation and question design, the first version of the survey scale was obtained. The initial survey scale was distributed, and 100 samples were collected for analysis and testing. The final scoring scale was obtained after modification.

This questionnaire collection adopts the form of face-to-face filling in or online contacting the research subjects to fill in the questionnaire. In order to ensure the consistency of the scale survey object and the word frequency statistical samples, the research objects of this scale survey are the customers of the six stores of CoCo mentioned above. We visited each store to distribute survey questionnaires or contacted online users of CoCo and sent the questionnaire link. A total of 170 questionnaires were distributed and 155 questionnaires were collected, with a questionnaire rate of 91.17%. After excluding 25 invalid questionnaires, a total of 130 valid questionnaires were collected. The average score of scale item of each element was calculated, and the statistical results are shown in Table 2.

**TABLE 2:** Scoring scale statistics table.

Domain	Item score	X
Money	Commodity prices are cheap 2.98; great offers and discounts 2.47; cost-effective 3.04; often use coupons to buy 1.71; often use points to deduct purchases 1.30; overall impression of money element 3.07	2.43
Commodity	The drink tastes good 3.38; rich drink ingredients 3.29; rich drink ingredients 3.25; variety of drinks 3.43; variety changes are always new 3.12; overall impression of commodity element 3.40	3.31
Service	Overall impression of information 3.66; lots of people in line and long waiting time 2.87; tables and chairs for dine-in/rest 3.20; insufficient manpower and slow order delivery 2.76; there are many takeaway orders in the store 3.22; I often order online 3.12; overall impression of service 3.54	3.20
Information	I often see promotion information 2.28; I often participate in group buying activities 2.05; I often participate in group buying activities 3.05; friends around will recommend CoCo’s drinks 2.78; overall impression of information 3.19	2.67
Value	Provide a sense of leisure and enjoyment 3.22; drinks are cool in summer and warm in winter, comfortable in season 3.38; CoCo is a popular brand 3.49; the shop is large and well-decorated 3.19; the shop is large and well-decorated 3.27; overall impression of value 3.38	3.32
Emotion	I like drinking CoCo 3.00; I feel happy when drinking CoCo 2.74; I have been drinking CoCo for many times 2.64; I would recommend CoCo’s drinks to my friends 2.68; overall impression of emotion 3.17	2.85
Status	CoCo’s business is good 3.58; CoCo has many shops 3.64; CoCo has been operating for a long time 3.52; for high consumers 2.86; high competitive position among similar beverage brands 3.16; overall impression of status 3.35	3.35

### Mathematical Expectation Prediction

#### Expectation Prediction Calculation

The goal of this stage is to calculate the *E(X)* and the *UPE*. Based on the classified word frequency statistics data and scoring scale statistics data, *X* and *P* of seven elements have been obtained. The corresponding data were used in the formula and the results were obtained as shown in Table 3: $E\,\left(X\right)\approx 3.09,\bar{X}\approx 3.02,$ the expectation value is slightly larger than the average evaluation score, which indicates that there are unsatisfied implicit expectations in the data of this case.

**TABLE 3:** Expectation prediction data statistics.

#	Money	Commodity	Service	Information	Value	Emotion	Status	AVG
X	2.43	3.31	3.2	2.67	3.32	2.85	3.35	3.02
P	4**.0**8%	39.98%	8.62%	8.45%	6.09%	26.83%	5.94%	–
UPE = X/P	59.56	8.28	37.12	31.60	54.52	10.62	56.40	36.87
E(X) = X_1_ × P_1_ + X_2_ × P_2_ + X_3_ × P_3_ + X_4_ × P_4_ + X_5_ × P_5_ + X_6_ × P_6_ + X_7_ × P_7_ = 3.09								

#### Expectation Prediction Interval Graph

**FIGURE 10:**
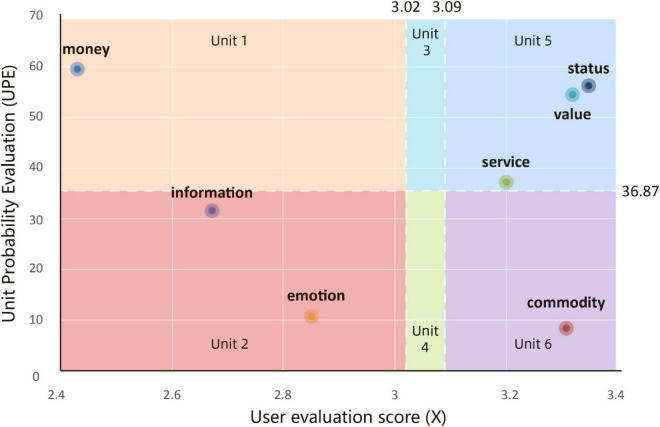
Expectation prediction interval graph of CoCo Fresh Tea & Juice.

Since $E\,\left(X\right)>\bar{X}$, the implicit expectation is unsatisfied, the improvement priority of the six intervals can be summarized from high to low as follows: Unit 2, Unit 1, Unit 4, Unit 3, Unit 6, and Unit 5. Among them, Unit 2 needs to be improved most. Units 6 and 5 do not need to be improved temporarily because their user satisfaction has reached the expectation value. The distribution of the seven exchange element nodes in the expectation prediction interval diagram is shown in Figure 10. The evaluation score of money is the lowest, while the higher ones are commodity, value, and status. The money, status, and value elements have higher *UPE*, while commodity and information are lower. It can be seen that the improvement of the information element and emotion element in Unit 2 has a necessity for improvement, while the user satisfaction of the information element has greater room for improvement. The money element in Unit 1 has a lot of room for improvement, but the least necessity for improvement is in user reviews (*UPE* is the highest). The evaluation scores and *UPE* of the status and value elements are relatively high, indicating that the user pays less concern to them and has reached a certain degree of satisfaction, and the commodity and evaluation scores of service elements also have exceeded expectation value. Therefore, there is no need to improve them temporarily. Table 4 represents the improvement priority of the exchange element and the extracted improvement items. At the same time, the diagram shows that no node falls in the implicit expectation interval, but referring to Table 2, it is found that the evaluation scores of the “Mobile applet ordering is more convenient” item of the information element and the “Cost-effective” item of the money element are in this interval. Improving these two items can not only meet implicit expectations but may also allow companies to get unexpected user praise.

**TABLE 4:** The results for extracted expectation.

No.	Exchange elements	Extract items that not meet user’s explicit/implicit expectations	Examples of solutions
1	Information	I often participate in group buying activities (2.05) I often see promotion information (2.28) Friends around will recommend CoCo’s drinks (2.78) Mobile applet ordering is more convenient (3.05, implicit)	Increasing the promotion of information about preferential activities such as group buying
2	Emotion	I have been drinking CoCo for many times (2.64) I would recommend to my friends (2.68) I feel happy when drinking CoCo (2.74) I like drinking CoCo (3.00)	Increase the loyalty of old users
3	Money	Often use points to deduct purchases (1.30) Often use coupons to buy (1.71) Great offers and discounts (2.47) Commodity prices are cheap (2.98) Cost-effective (3.04, implicit)	Recommend users to use points or coupons for settlement
4	Service	Insufficient manpower, slow order delivery (2.76) Lots of people in line and long waiting time (2.87)	Improve service efficiency and reduce waiting time
	Commodity	None	None
	Value	None	None
	Status	For high class of consumers (2.86)	None (because this is determined by CoCo’s brand positioning).

The expectation prediction results show that information, emotion, and money are the priority items to be improved. In order to further test and verify the validity of the data, we applied paired sample *t*-test in SPSS^2^ software to statistically analyze the difference in information, emotion, and money. As shown in Table 5, the results showed a statistically significant difference (p<0.05).

**TABLE 5:** Paired sample *t*-test.

No.	Paired sample	*p*
1	X (money) – X (information)	0.003
2	X (money) – X (emotion)	0.000
3	X (information) – X (emotion)	0.029
4	UPE (money) – UPE (information)	0.000
5	UPE (money) – UPE (emotion)	0.000
6	UPE (information) – UPE (emotion)	0.000

## Discussion

The results of this study show that, compared with the optimization and improvement in commodity and service elements, the improvement in information, emotion, and money elements can improve user satisfaction and optimize the consumption experience more effectively. The results also conform to the consumption development trend under the background of the digital service economy. For example, information elements such as live-stream shopping (Sun et al., 2019; Ma, 2021) and festival discount activities (Sozer, 2019; Sung, 2020; Zane et al., 2021) have a great impact on promoting consumption behavior.

Facing the two challenges proposed in section “Introduction,” the expectation prediction framework and analysis method proposed in this study have the following advantages: (1) The examples in this study collect large-scale text data, and data-driven technology improves the efficiency and accuracy of user-evaluation data analysis and demand mining as well as improves the replicability of the expectation prediction method. (2) Website review data and user evaluation data have played a role in supplementing and verifying the conceptual definition of exchange elements, making the construction of the seven conceptual areas more complete and more suitable for the complex and changeable characteristics of requirements of consumers. If the social environment background or social exchange resources have changed or need to be redefined, the framework can still guide the expected prediction of social exchange elements. (3) Social exchange theory has unique perspectives, thinking, and methods and can contribute to a comprehensive and clear understanding of the consumption process of various aspects such as exchange resource classification, user behavior, and attitude, and exchange relationships provide a theoretical framework of user demand mining. Despite the above advantages, this study still has some limitations: (1) Due to the limitation of data acquisition, this study only crawled user-generated data from an open comment website in China, so it only verified the effectiveness of this method for Chinese comment data. (2) This study has not yet realized an automatic and complete data crawling analysis software. The manual classification process of user comment data is not accurate enough and has errors. (3) Lacking further discussion on the correlation between various exchange elements, such as the influence of other exchange elements on emotional elements, the correlation between information elements and monetary elements, and so on. In general, these limitations do not affect the verification process of the expected prediction framework based on social exchange resources.

## Conclusion

The development of information technology, such as the Internet, big data, and cloud computing, has given birth to the digital economy. It takes stakeholders as participants in co-creation, realizes the rapid optimization and regeneration of resources through a large amount of data analysis, and meets user needs continuously and sustainably. In this complex system, the innovative use of massive data plays a vital role in design innovation. Therefore, effectively tapping the hidden needs of stakeholders has become a crucial step. This project introduces a new data-driven framework to support the expectation prediction process of social exchange resources. The main contributions of this research can be summarized in three aspects:

(1) Apply social exchange theory to the process of user comment data mining. Based on the social exchange theory, expectation prediction is different from the conventional demand analysis process in two aspects: (a) data-driven under the digital service economy and (b) the classification and concept definition of consumption exchange elements of which the classification and statistics of user evaluation data are better guided.
(2) A data-driven method combining word frequency statistics and scale surveys is proposed. Word frequency statistics analyze large-scale user comment data, and evaluation scales quantitatively investigate a small sample of stakeholders. The two methods complement each other and replace the conventional requirement analysis method, which provides a new perspective for demand analysis engineering.
(3) Define an expectation prediction interval graph based on mathematical expectation analysis. According to the mathematical expectation value, arithmetic average value, and the average value of UPE, seven exchange elements are divided in the expectation prediction interval diagram, and the improvement priority of exchange elements is defined, thereby improving the efficiency of expectation prediction.

In addition, the example of the design improvement in the CoCo Fresh Tea & Juice service system was further adopted to verify the feasibility of the data-driven expectation prediction framework. The author hopes this framework can be seen as the foundation for supporting expectation forecasts and further value co-creation process in the context of the digital service economy. At the same time, it is recommended to conduct more in-depth research in the future to (1) investigate user comments in other languages, further verify the feasibility of the framework, and define a more comprehensive and standardized concept of social exchange elements and (2) consider and increase cross-predictive analysis on various exchange elements and explore the correlation between elements.

## Data Availability Statement

The original contributions presented in the study are included in the article/supplementary material, further inquiries can be directed to the corresponding author.

## Ethics Statement

Written informed consent was obtained from the individual(s) for the publication of any potentially identifiable images or data included in this article.

## Author Contributions

EC developed the ideas, logically arranged them, and revised the manuscript. JJ supported in writing, logical arrangement of ideas, and implementation. YD performed statistical analysis and review. HP supervised and reviewed the manuscript. All authors contributed to the article and approved the submitted version.

## Conflict of Interest

The authors declare that the research was conducted in the absence of any commercial or financial relationships that could be construed as a potential conflict of interest.

## Publisher’s Note

All claims expressed in this article are solely those of the authors and do not necessarily represent those of their affiliated organizations, or those of the publisher, the editors and the reviewers. Any product that may be evaluated in this article, or claim that may be made by its manufacturer, is not guaranteed or endorsed by the publisher.

## Footnotes

1
https://www.gooseeker.com/

2
https://www.ibm.com/cn-zh/products/spss-statistics
